# Genomic characterization and niche adaptive analysis of *Pseudomonas promysalinigenes* W2469: the first clinical isolate from a human bile specimen

**DOI:** 10.3389/fcimb.2026.1825480

**Published:** 2026-05-18

**Authors:** Shan Zhong, Lei Xing, Huan Li, Licheng Wang, Xiong Zhu, Chongzhen Wang

**Affiliations:** 1 Clinical and Central Laboratory of Sanya People’s Hospital, Sanya, Hainan, China; 2 Department of Hematology and Oncology of Sanya People’s Hospital, Sanya, Hainan, China

**Keywords:** antimicrobial resistance homologous genes, bacterial genomic diversity, bile specimen, first clinical isolate, MALDI-TOF MS, niche adaptation, *Pseudomonas promysalinigenes*, whole genome sequencing (WGS)

## Abstract

**Background:**

*Pseudomonas promysalinigenes* is a newly described bacterial species renowned for producing promysalin, a species-selective lipopeptide antibiotic. All previously reported strains of this species are derived from environmental niches such as plant rhizospheres, and no clinical infection cases associated with this bacterium have been documented to date. Thus, the clinical microbiology relevance, genomic features, and adaptive potential of *P. promysalinigenes* remain largely unexplored, and current reference databases have limited coverage of this rare species.

**Methods:**

A bacterial strain designated W2469 was isolated from the bile specimen of a patient with acute suppurative cholecystitis and cholecystolithiasis. Conventional phenotypic and molecular identification [matrix-assisted laser desorption/ionization time-of-flight mass spectrometry (MALDI-TOF MS), VITEK 2 biochemical assay, 16S ribosomal RNA (rRNA), and whole-genome sequencing (WGS)] was performed. Bioinformatics analyses, including average nucleotide identity (ANI), digital DNA–DNA hybridization (dDDH), core genome single-nucleotide polymorphism (cgSNP), pan-genome analysis, and functional annotation against COG, KEGG, CAZy, VFDB, and CARD databases, were conducted to characterize the strain.

**Results:**

Conventional methods yielded consistent misidentification of the strain, while WGS definitively assigned it to *P. promysalinigenes* (ANI = 98.8%, dDDH = 91.1% against the type strain RW10S1). The strain exhibited a narrow-spectrum resistance phenotype, with resistance to aztreonam and ticarcillin/clavulanic acid, intermediate susceptibility to meropenem, and susceptibility to most clinically used antibiotics. Genomic annotation identified 25 antimicrobial resistance genes and 139 niche adaptation-related factors, most of which are low-identity homologs (<80%) of canonical reference sequences. Pan-genome analysis identified 571 clinical-specific genes associated with host adaptation, with complete loss of the environmental promysalin biosynthetic gene cluster.

**Conclusion:**

This study provides the first documentation of *P. promysalinigenes* as a clinical isolate from human bile, expanding the known ecological niche of this species to the clinical setting. Conventional methods are prone to misidentifying this rare species, and WGS is critical for accurate taxonomic identification. Importantly, the strain exhibits clear adaptive phenotypes despite low sequence identity to known functional elements, highlighting profound knowledge gaps in the genomic diversity and uncharacterized adaptive mechanisms of this rare *Pseudomonas* species. This work provides a foundational genomic resource for future investigations into this emerging opportunistic pathogen.

## 1 Introduction

The genus *Pseudomonas* comprises Gram-negative, aerobic, flagellated, non-spore-forming bacilli with extraordinary species diversity, and these bacteria are widely distributed in natural environments and the human microecosystem (Gomila et al., 2015; Peix et al., 2018). The genus *Pseudomonas* has extraordinary species diversity, with most species originating from natural environmental niches. In recent years, with the popularization of whole-genome sequencing (WGS), an increasing number of rare environmental *Pseudomonas* species have been detected in clinical specimens, revealing the phenomenon of environmental bacteria adapting to clinical niches (Qin et al., 2022; Yi and Dalpke, 2022; Tohya et al., 2022; Arbefeville et al., 2024). However, the adaptive mechanisms of these rare species from environmental to clinical settings remain largely unexplored. However, overlapping phenotypic and biochemical characteristics between these rare species and common clinically relevant *Pseudomonas* species frequently cause misidentification by conventional methods such as MALDI-TOF MS and biochemical assays. This is mainly attributed to the incomplete coverage of characteristic spectra and biochemical reaction profiles of rare species in commercial databases (Arbefeville et al., 2024; Udaondo et al., 2024). 16S rRNA sequencing, a gold-standard tool for preliminary bacterial identification, also has inherent limitations in distinguishing closely related *Pseudomonas* species because of the high conservation of the 16S rRNA gene (Klindworth et al., 2013; Church et al., 2020).

WGS has emerged as a high-resolution and reliable tool for the accurate taxonomic identification of rare and newly described pathogenic bacteria (Rudra and Gupta, 2024; Saati-Santamaría et al., 2021). Combined with genomic analyses such as average nucleotide identity (ANI), digital DNA–DNA hybridization (dDDH), and core genome single-nucleotide polymorphism (cgSNP) phylogenetic tree construction, WGS can achieve definitive species delineation that cannot be achieved by conventional methods. In addition, WGS supports comprehensive genomic characterization of bacterial virulence factors, antimicrobial resistance mechanisms, and environmental adaptability, which is critical for understanding the clinical significance and pathogenic potential of newly identified clinical isolates (Rudra and Gupta, 2024).

*Pseudomonas promysalinigenes* was validly published and correctly named in 2022 in the List of Prokaryotic Names with Standing in Nomenclature (LPSN) database, and this species was previously classified under *Pseudomonas putida* (Migula, 1895). The type strain RW10S1 was isolated from rice rhizosphere in Sri Lanka and formally described in 2021. This species has attracted extensive research attention because of its unique secondary metabolite promysalin—a lipopeptide antibiotic that selectively inhibits the growth of *Pseudomonas aeruginosa* while being non-toxic to the producing strain itself (Li et al., 2011; Das et al., 2023). Another environmental isolate, RL-WG26, derived from the rhizosphere of salt-tolerant rice, has been shown to promote plant growth and alleviate salt stress, revealing its genetic basis as a plant growth-promoting rhizobacterium (Ren et al., 2024).

To date, no clinical infection cases related to *P. promysalinigenes* have been reported worldwide, and it remains unknown whether this bacterium can colonize the human body, adapt to the human microenvironment, and cause infectious diseases. This critical knowledge gap poses three major challenges to clinical microbiology practice: 1) Conventional identification methods may misidentify this rare species due to incomplete coverage in commercial databases, leading to delayed or incorrect diagnosis; 2) the lack of information on its antimicrobial resistance profile may result in inappropriate empirical antibiotic therapy; and 3) the genomic basis of niche adaptation from the plant rhizosphere to the human host remains completely unexplored, hindering our understanding of the ecological expansion of environmental *Pseudomonas* species. Therefore, in this study, we isolated a *P. promysalinigenes* strain (designated W2469) from the bile specimen of a 54-year-old male patient with acute suppurative cholecystitis and cholecystolithiasis—the first reported clinical isolate of this species worldwide. The primary objectives of this study were 1) to systematically evaluate the performance of conventional clinical identification methods for this rare species; 2) to characterize the genomic features of the first clinical isolate, including antimicrobial resistance and host adaptation-related genomic elements; 3) to explore the adaptive evolution of this environmental species to the human host niche via pan-genome analysis; and 4) to highlight the limitations of current reference databases for annotating rare non-fermenting bacteria. This study aims to fill critical knowledge gaps regarding the clinical microbiology relevance of *P. promysalinigenes*, and provide a foundational genomic resource for understanding the broader diversity of the *Pseudomonas* genus.

## 2 Case presentation

**Figure 1:**
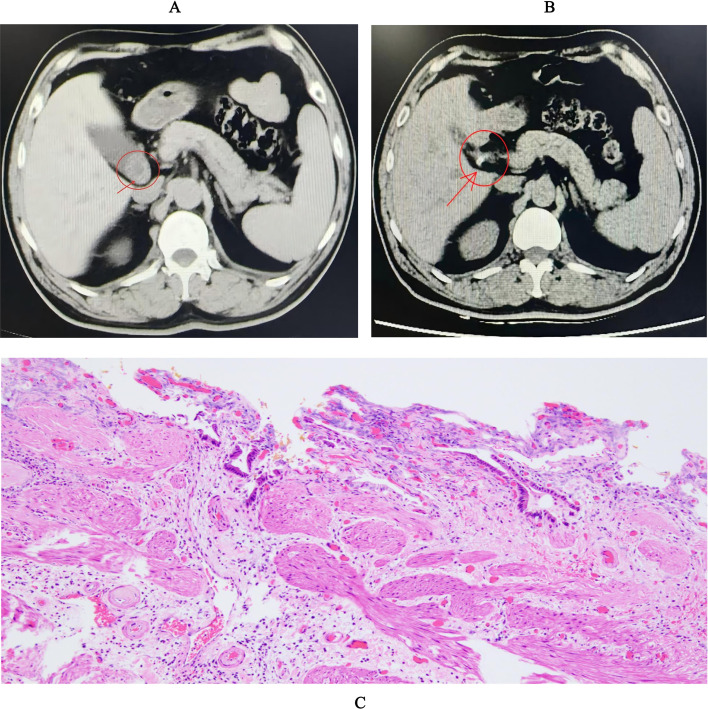
Clinical and pathological findings of the patient. **(A)** Abdominal CT showing gallstones with cholecystitis (pointed by the red arrow: a nodular mixed high-density lesion was identified in the region of the gallbladder neck). **(B)** After laparoscopic cholecystectomy (pointed by the red arrow: absence of the gallbladder). **(C)** Pathological section of gallbladder tissue showing acute suppurative cholecystitis with cholelithiasis (HE staining, ×100).

On 1 March 2025, a 54-year-old male patient was admitted to the Emergency Department of Sanya People’s Hospital due to spontaneous paroxysmal colic in the right upper abdomen radiating to the right shoulder. The symptom occurred without obvious inducement, and no accompanying symptoms (e.g., nausea, vomiting, chills, fever) were observed. Oral cholagogic and anti-inflammatory drugs showed no therapeutic effect. Abdominal computed tomography (CT) revealed cholecystolithiasis complicated with cholecystitis (**Figure 1A**), and the patient was emergently admitted to the Department of General Surgery with a preliminary diagnosis of cholecystolithiasis complicated with cholecystitis.

Physical examination on admission showed the following vital signs: body temperature 36.5 °C, pulse rate 63 beats/min, respiratory rate 20 breaths/min, and blood pressure 118/66 mmHg. Abdominal examination showed a soft abdominal wall, tenderness in the right upper abdomen, and a positive Murphy’s sign. The patient had no history of hypertension, diabetes mellitus, coronary heart disease, or other chronic diseases. Hepatobiliary color Doppler ultrasound further confirmed cholecystolithiasis complicated with cholecystitis, and a possible small cyst was found in the left lateral segment of the liver. The final admission diagnoses were acute cholecystitis complicated with cholecystolithiasis and a small cyst in the left lateral segment of the liver.

Immediate blood test results were as follows: white blood cell count 7.77 × 10^9^/L, neutrophil ratio 87.7% (normal range: 40%–75%), high-sensitivity C-reactive protein 53 mg/L (normal range: 0–5 mg/L), and hemoglobin 126 g/L (normal range: 131–172 g/L). Liver function indicators were as follows: serum alanine aminotransferase 58 U/L (normal range: 9–50 U/L), total bilirubin 27.3 μmol/L (normal range: 0–26 μmol/L), direct bilirubin 9.5 μmol/L (normal range: 0.3–6.8 μmol/L), and indirect bilirubin 17.8 μmol/L (normal range: 1.7–17.0 μmol/L).

Based on the patient’s clinical manifestations, imaging findings, and laboratory results, laparoscopic cholecystectomy was performed (**Figure 1B**). During surgery, sterile fine−needle aspiration was performed directly from the gallbladder lumen under full laparoscopic visualization, before gallbladder wall incision to collect bile for microbiological examination. Strict aseptic techniques were used throughout the collection to avoid skin or environmental contamination. The bile sample was immediately transferred to the clinical microbiology laboratory in a sealed sterile container, and a gallbladder tissue section was submitted for pathological examination. The clinical microbiology laboratory reported isolation of a *Pseudomonas* species from the bile culture, which was finally identified as *P. promysalinigenes* by whole-genome sequencing. Pathological examination of the gallbladder tissue was consistent with acute suppurative cholecystitis complicated with cholelithiasis (**Figure 1C**). The patient received postoperative anti-infective treatment with piperacillin for 1 week, and clinical symptoms improved significantly; the patient then requested voluntary discharge.

## 3 Materials and methods

### 3.1 Bile culture and bacterial isolation

The bile specimen collected intraoperatively was immediately transported to the clinical microbiology laboratory in a sterile container and processed in strict accordance with standard operating procedures of clinical microbiological testing. The bile specimen was inoculated onto Columbia blood agar plates (Autobio Diagnostics Co., Ltd., China), and a direct smear of the bile specimen was prepared for Gram staining and microscopic examination. Inoculated plates were incubated aerobically at 35 °C in a 5% CO_2_ incubator for 24–48 h, and colony growth was observed daily. Single bacterial colonies with uniform morphology were selected and purified by repeated streaking on Columbia blood agar plates for subsequent identification and experimental analysis.

### 3.2 Bacterial phenotypic identification

#### 3.2.1 MALDI-TOF MS identification

Purified bacterial colonies were spotted onto a stainless steel MALDI-TOF MS target plate, mixed with 1 μL of α-cyano-4-hydroxycinnamic acid (CHCA) matrix solution (bioMérieux, France), and air-dried at room temperature. The target plate was loaded into the VITEK^®^MS automated mass spectrometry identification system (bioMérieux, France), and data acquisition and species identification were performed according to the manufacturer’s instructions. A confidence level ≥90% was considered a reliable species-level identification result, while a confidence level <90% was regarded as unreliable for species confirmation, in accordance with the manufacturer’s instructions and previous standard protocols (Seng et al., 2009).

#### 3.2.2 Biochemical identification

Biochemical characteristics of the purified isolate were determined using the VITEK 2 GN identification card (bioMérieux, France) on the VITEK 2 compact automated microbial analysis system (bioMérieux, France). The card was inoculated with the bacterial suspension and loaded into the instrument. The instrument automatically performed biochemical reactions, recorded results, and calculated the species identification rate. An identification rate ≥95% was defined as reliable species-level biochemical identification, in accordance with the standard operating procedures of the VITEK 2 system and previous clinical microbiology guidelines (Renaud et al., 2005; Versalovic et al., 2021).

### 3.3 Antimicrobial susceptibility testing

Antimicrobial susceptibility testing of the isolated strain was performed using the VITEK 2 compact automated microbial analysis system (bioMérieux, France) with the corresponding susceptibility card, in strict accordance with the Clinical and Laboratory Standards Institute (CLSI) M100-S35 standard for non-fermenting Gram-negative bacilli. *Pseudomonas aeruginosa* (ATCC 27853) was used as the quality control strain to determine the minimum inhibitory concentrations (MICs) of the antimicrobial agents for W2469, and the results were interpreted as susceptible (S), intermediate (I), or resistant (R) based on the CLSI M100-S35 breakpoints for *Pseudomonas* species (Clinical and Laboratory Standards Institute, 2025).

### 3.4 Genomic DNA extraction and 16S rRNA sequencing

Genomic DNA was extracted from an overnight liquid culture of the purified isolate (Luria–Bertani broth, 35 °C, 180 rpm shaking culture) using the TIANamp Bacterial DNA Kit (TIAGEN BIOTECH, China) in strict accordance with the manufacturer’s protocol. The concentration and purity of the extracted genomic DNA were detected using a NanoDrop spectrophotometer, and integrity was verified by 1% agarose gel electrophoresis.

The 16S rRNA gene of the isolate was amplified by polymerase chain reaction (PCR) using the universal bacterial primers 27F (5′-AGAGTTTGATCCTGGCTCAG-3′) and 1492R (5′-GGTTACCTTGTTACGACTT-3′). PCR amplification and product purification were performed by RuiBio BioTech Co., Ltd. (Beijing, China), and Sanger sequencing was performed to obtain the 16S rRNA gene sequence. This sequence was deposited in the NCBI GenBank database under accession number PX899384. The sequencing results were uploaded to the NCBI BLAST database for nucleotide homology comparison, and bacterial species with a sequence similarity ≥99% were selected as candidate identification results (Yoon et al., 2017).

### 3.5 Whole-genome sequencing and assembly

Whole-genome sequencing of the purified isolate W2469 was performed by BIOYI GENE Tech Co., Ltd. (Wuhan, China) using the DNBSEQ-T7 high-throughput sequencing platform (MGI, China), which generated 150 bp paired-end reads (Li et al., 2025). Raw sequencing data were filtered using fastp v0.23.4 with the following parameters: -q 20 -u 20 -n 5 -I 50, to remove low-quality reads, adapter sequences, and contaminated sequences, yielding high-quality clean data. Quality control of the clean data was performed using FastQC (http://www.bioinformatics.babraham.ac.uk/projects/fastqc) to evaluate the sequencing quality.

The clean data were assembled and corrected using Unicycler (parameters: -1 r1–2 r2 --keep 3 --mode normal) to obtain the final genome sequence (Wick et al., 2017). Basic genomic features, including genome size, number of contigs, contig N50 value, and GC content, were statistically analyzed.

The raw whole-genome sequencing reads and the assembled genome sequence of strain W2469 were deposited in the NCBI SRA and NCBI GenBank databases under accession numbers SRR38011177 and PRJNA1397510, respectively.

### 3.6 Genomic taxonomic identification

To confirm the taxonomic affiliation of strain W2469, ANI and dDDH analyses were performed between W2469 and the *P. promysalinigenes* type strain RW10S1 (GenBank accession: GCF_014269025.2). ANI was calculated using FastANI v1.3 with a minimum alignment fraction of 0.5 (Kim et al., 2014), and dDDH was analyzed using the online Genome-to-Genome Distance Calculator (GGDC 3.0, https://ggdc.dsmz.de/) with Formula 2 (Goris et al., 2007). Species delineation thresholds were ANI ≥95% and dDDH ≥70% for the same bacterial species.

cgSNP phylogenetic tree construction was performed using Snippy v4.6.0 with the following parameters: --mincov 10 --minqual 30, to extract core SNP loci, with recombination regions removed by Gubbins v3.2.1. Genomic sequences of W2469 and reference *Pseudomonas* strains were imported into Snippy v4.6.0, with the *P. promysalinigenes* type strain RW10S1 (GCA_014269025.2) as the reference to extract core SNP loci. The phylogenetic tree was constructed using FASTTREE v2.1.11 based on the maximum likelihood (ML) method with the GTR+CAT nucleotide substitution model and 1,000 bootstrap replicates. The phylogenetic tree was visualized and annotated using the Interactive Tree Of Life (iTOL, https://itol.embl.de/), and the taxonomic status of strain W2469 was determined based on phylogenetic clustering.

### 3.7 Functional genomic annotation and pathogenicity analysis

The assembled genomic sequence of strain W2469 was subjected to comprehensive functional annotation and pathogenicity-related analysis using multiple international public databases. Detailed methods are as follows:

COG and KEGG annotation: Clusters of Orthologous Groups (COG) database annotation was performed to classify protein-coding genes into different functional categories (Galperin et al., 2015); Kyoto Encyclopedia of Genes and Genomes (KEGG) database annotation was used to analyze metabolic pathways and their association with human diseases (Kanehisa et al., 2014).

CAZy annotation: Carbohydrate-Active enZYmes (CAZy) database annotation was conducted to identify and classify genes encoding carbohydrate-active enzymes in the genome (Park et al., 2010; Cantarel et al., 2009).

Virulence factor prediction: Pathogenic Bacteria database (VFDB, core dataset; Chen et al., 2005) was used to predict niche adaptation-related homologous factors via BLASTp with strict thresholds: e-value ≤1e−10, amino acid identity ≥40%, and query coverage ≥50%. Positive hits were classified by functional characteristics.

Antibiotic resistance gene prediction: The Comprehensive Antibiotic Resistance Database (CARD, v3.2.4; Alcock et al., 2020) was used to identify AMR homologous genes via the Resistance Gene Identifier (RGI v 6.0.5) strict model, with a minimum 50% amino acid identity and 50% query coverage threshold. Resistance mechanisms and associated antibiotic classes were annotated for each positive hit. In parallel, the porin protein *OprD* of W2469 was predicted using PorinPredict (95% amino acid identity threshold) (https://github.com/MBiggel/PorinPredict).

Prediction of mobile genetic elements (MGEs): Prophages, genomic islands (GIs), plasmids, and insertion sequences in the W2469 genome were predicted using Prophage-DB (Dieppa-Colón et al., 2025, https://github.com/etandieppa/Prophage-DB), IslandPath-DIMOB v1.0.0 (Bertelli and Brinkman, 2018), PlasmidFinder v 2.1.6 (Carattoli and Hasman, 2020), and the ISfinder v 2.0 database (Siguier et al., 2012), respectively. Subsequently, genomic sequences of the predicted MGEs were then aligned and annotated against (identity ≥80%, coverage ≥60%) the ResFinder v 4.7.2 and VirulenceFinder v 2.0 databases to further examine the location of antimicrobial resistance genes and virulence factors on these elements in W2469. This analysis aimed to assess the potential dissemination of resistance genes and adaptive genomic evolution of this rare pathogen.

T3SS effector protein prediction: Genes encoding type III secretion system (T3SS) effector proteins, a key virulence system in Gram-negative pathogens, were identified using the Effective T3 online tool (https://github.com/nicolasrnemeth/EffectiveT3) (Arnold et al., 2009).

Pan-genome analysis and clinical-specific adaptive genes: Pan-genome analysis was performed using Roary v3.13.0 with a 95% BLASTp identity threshold for core gene clustering, enabling comparison of the clinical isolate *P. promysalinigenes* W2469 with three environmental strains (RW10S1: GCA_014269025.2, RL-WG26: GCA_025398015.1, KZH.chiA: GCA_046267955.1) isolated from rice rhizosphere (Page et al., 2015). This approach aimed to identify clinical-specific adaptive genes distinct from those associated with environmental factors. Then, functional annotation of genes unique to clinical strain W2469 was performed to identify “clinically specific” genes that may contribute to adaptation to the human host, in contrast to the typical rice rhizosphere niche.

Promysalin biosynthetic gene cluster analysis: Systematic secondary metabolite biosynthetic gene cluster (BGC) prediction for strain W2469 was performed using antiSMASH v8.0 (Blin et al., 2025) with a relaxed detection mode and a minimum 60% sequence identity threshold for biosynthetic gene cluster annotation. BLASTn alignment was further performed against the full promysalin BGC sequence of the environmental type strain *P. promysalinigenes* RW10S1.

## 4 Results

### 4.1 Phenotypic characteristics and preliminary identification of strain W2469

**Figure 2:**
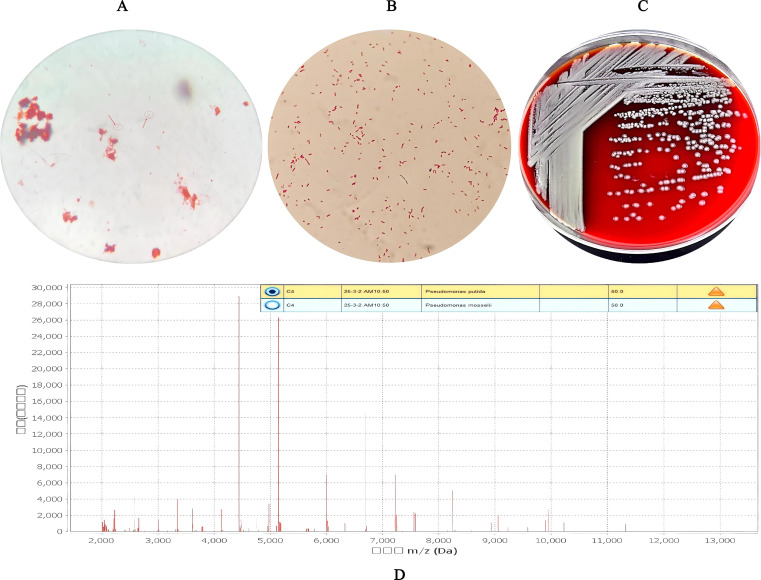
Phenotypic characteristics and preliminary identification of *Pseudomonas promysalinigenes* W2469. **(A)** Gram staining of original bile smear (×1,000) (pointed by the red arrow). **(B)** Gram staining of purified isolate (×1,000). **(C)** Colonies on Columbia blood agar (35 °C, 5% CO_2_, 24 h). **(D)** MALDI-TOF MS identification results.

Gram staining of both the original bile smear and the purified bacterial isolate revealed Gram-negative bacilli with short rod-shaped morphology (**Figures 2A, B**). After 24 h of incubation on Columbia blood agar plates at 35 °C in a 5% CO_2_ atmosphere, strain W2469 formed round, convex, white, smooth, moist colonies with a uniform diameter of 1–2 mm (**Figure 2C**), and no hemolysis was observed on the blood agar.

Preliminary identification by MALDI-TOF MS showed that strain W2469 had a 50% confidence match with both *Pseudomonas putida* and *Pseudomonas mosselii*, with no dominant species match (**Figure 2D**), which did not meet the reliable identification standard (confidence level ≥90%). In contrast, biochemical identification using the VITEK 2 GN card classified the strain as *P. putida* with a 99% identification rate, revealing a clear discrepancy between the two phenotypic methods. Detailed biochemical reaction characteristics of strain W2469 detected by the VITEK 2 compact system are listed in **Table 1** (e.g., positive for GGT, ProA, SUCT; negative for APPA, H2S, BGLU).

**Table 1:** The biochemical reaction characteristics of *Pseudomonas promysalinigenes* W2469.

Item	Value	Item	Value	Item	Value
APPA	−	PyrA	−	dCEL	−
H_2_S	−	AGLTp	−	GGT	+
BGLU	−	dMAN	−	BXYL	−
ProA	+	PLE	−	URE	−
SAC	−	dTRE	−	MNT	+
ILATk	−	SUCT	+	AGAL	−
GlyA	−	LDC	−	CMT	+
O129R	+	IMLTa	+	ILATa	+
ADO	−	IARL	−	BGAL	−
BNAG	−	dGLU	+	OFF	−
dMAL	−	dMNE	+	BAlap	−
LIP	−	TyrA	+	dSOR	−
dTAG	−	CIT		5KG	−
AGLU	−	NAGA	−	PHOS	−
ODC	−	IHISa	+	BGUR	−
GGAA	−	ELLM	−		

The biochemical test was performed using the VITEK 2 GN card supplied with the VITEK 2 compact automated microbial identification system.

+, positive; −, negative.

### 4.2 Antimicrobial susceptibility profile of strain W2469

Antimicrobial susceptibility testing results showed that strain W2469 was susceptible to most clinical antibiotics used to treat *Pseudomonas* infections, including piperacillin/tazobactam, gentamicin, tobramycin, ceftazidime, levofloxacin, ciprofloxacin, amikacin, cefepime, imipenem, and piperacillin (**Table 2**). The strain was resistant to aztreonam (MIC ≥64 μg/mL) and ticarcillin/clavulanic acid (MIC ≥128/2 μg/mL) and showed intermediate susceptibility to meropenem (MIC = 4 μg/mL). No multidrug resistance (MDR) phenotype was observed.

**Table 2:** The antimicrobial susceptibility test results of *Pseudomonas promysalinigenes* W2469.

Antimicrobial agents	MIC (μg/mL)	Interpretation
Piperacillin/tazobactam	≤8/4	S
Gentamicin	≤2	S
Tobramycin	≤1	S
Ceftazidime	=4	S
Levofloxacin	=1	S
Aztreonam	≥64	R
Ciprofloxacin	≤0.25	S
Amikacin	≤4	S
Cefepime	=4	S
Imipenem	≤1	S
Meropenem	=4	I
Ticarcillin/clavulanic acid	≥128/2	R
Piperacillin	≤8	S

*In vitro* drug susceptibility test was performed by broth microdilution method in strict accordance with the CLSI M100-S35 standard (MIC, minimum inhibitory concentration; S, sensitive; I, intermediate; R, resistant).

### 4.3 16S rRNA sequencing analysis

Sanger sequencing of the 16S rRNA gene of strain W2469 generated a 1,410-bp high-quality sequence. NCBI BLAST homology comparison showed that this sequence shared 99.79% similarity with both *Pseudomonas urethralis* (GenBank accession: NR181197.1) and *Pseudomonas juntendi* (GenBank accession: NR180497.1). Because sequence similarity with both species was ≥99% with no significant difference, 16S rRNA sequencing could not determine the exact species of strain W2469, reflecting the inherent limitation of 16S rRNA sequencing in distinguishing closely related *Pseudomonas* species.

### 4.4 Genomic features of strain W2469

High-throughput whole-genome sequencing and *de novo* assembly of strain W2469 generated 48 contigs with a contig N50 of 325,916 bp. The total genome length of W2469 was 5,107,534 bp with a GC content of 60.45%, consistent with the genomic features of the fluorescent *Pseudomonas* species. The genome contained 4,865 genes, including 4,671 protein-coding genes (96.01% of total genes), 70 transfer RNA (tRNA) genes, and 5 ribosomal RNA (rRNA) genes (1 copy of 16S rRNA, 2 copies of 5S rRNA, and 2 copies of 23S rRNA). Detailed genomic features of strain W2469 are summarized in **Table 3**.

**Table 3:** The genomic features of *Pseudomonas promysalinigenes* W2469.

Type	Number
Genome size (bp)	5,107,534
Contig number	48
Contig N50 (bp)	325,916
G+C content (%)	60.45
Protein-coding genes (CDS)	4,671
tRNAs	70
16S rRNA	1
5S rRNAs	2
23S rRNAs	2

### 4.5 Genomic confirmation of taxonomic affiliation of strain W2469

ANI analysis between strain W2469 and the *P. promysalinigenes* type strain RW10S1 showed an ANI value of 98.8%, well above the 95% species. dDDH analysis using Formula 2 produced a dDDH value of 91.1%, far exceeding the 70% threshold for defining the same bacterial species.

**Figure 3:**
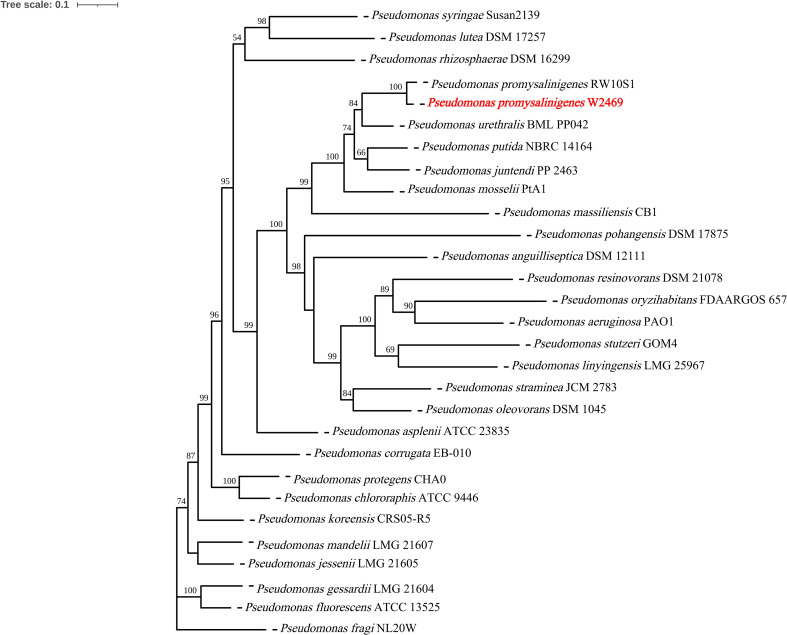
Core genome SNP (cgSNP) phylogenetic tree of *Pseudomonas promysalinigenes* W2469 and reference strains. The tree was constructed using the maximum likelihood method with bootstrap values (>50%) based on 1,000 replicates are shown at the branch nodes. W2469 is marked with a red text.

The cgSNP phylogenetic tree constructed with 1,000 bootstrap replicates showed that strain W2469 clustered tightly with the *P. promysalinigenes* type strain RW10S1 with 100% bootstrap support, forming an independent monophyletic branch. The strain was clearly separated from other closely related *Pseudomonas* species including *P. putida*, *P. mosselii*, *P. urethralis*, and *P. juntendi* (**Figure 3**). Based on ANI, dDDH, and cgSNP phylogenetic analyses, strain W2469 was definitively confirmed as *P. promysalinigenes*.

We accurately identified strain W2469 using WGS combined with ANI, dDDH, and cgSNP analyses. Results were compared with conventional clinical identification methods, including MALDI-TOF MS, the VITEK 2 GN card, and 16S rRNA gene sequencing (**Table 4**). Comparative evaluation showed that WGS has clear advantages and high accuracy in identifying rare and newly described *Pseudomonas* species. This study highlights the essential and irreplaceable value of WGS as the gold-standard method for identifying such pathogens.

**Table 4:** Comparison of conventional identification methods and WGS-based definitive identification of strain W2469.

Identification method	Result	Confidence/similarity	Misidentification type	Definitive ID by WGS
MALDI−TOF MS	*P. putida*/*P. mosselii*	50%	Low-confidence cross−match	*P. promysalinigenes*
VITEK 2 GN	*P. putida*	99%	High-confidence false ID	*P. promysalinigenes*
16S rRNA sequencing	*P. urethralis*/*P. juntendi*	99.79%	Closely related species blur	*P. promysalinigenes*
WGS (ANI + dDDH + cgSNP)	*P. promysalinigenes*	ANI = 98.8%, dDDH = 91.1%	Correct species delineation	*P. promysalinigenes*

ANI, average nucleotide identity; dDDH, digital DNA–DNA hybridization; WGS, whole−genome sequencing.

### 4.6 Functional genomic annotation of strain W2469

#### 4.6.1 COG and KEGG functional annotation

**Figure 4:**
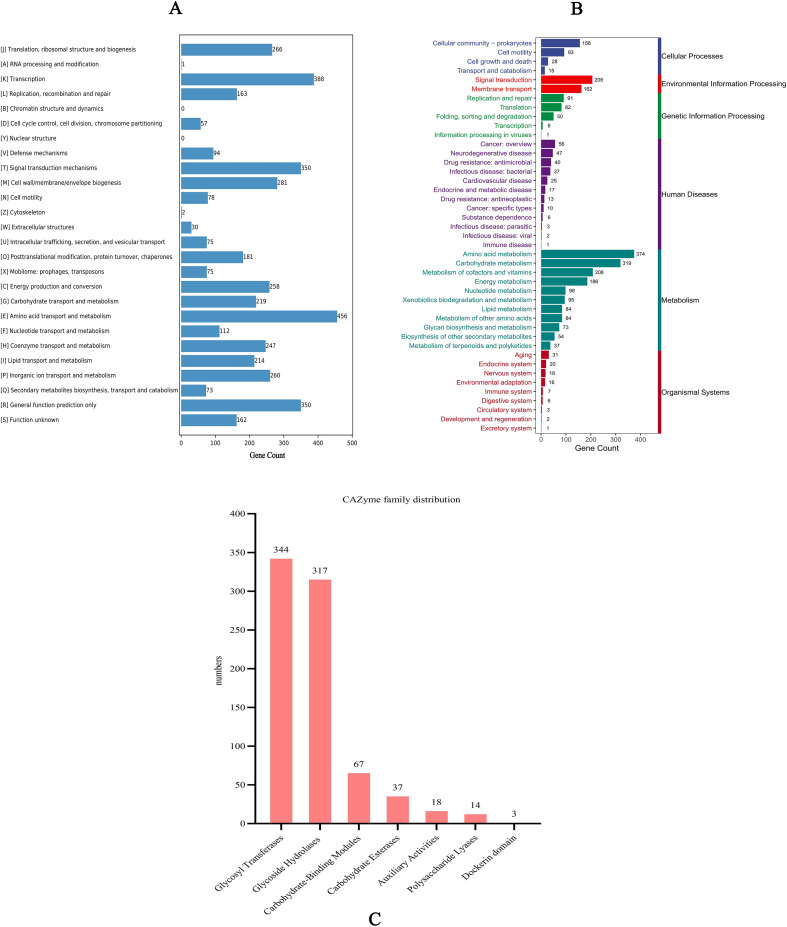
Functional annotation of *Pseudomonas promysalinigenes* W2469 genome. **(A)** COG functional annotations were grouped into 24 categories, mainly enriched in pathways such as metabolism, transcription, and signal transduction mechanisms; the *X*- and *Y*-axes indicate gene count and COG classification, respectively. **(B)** KEGG annotation was mapped to six different functional categories, mainly involving cellular processes, environmental information processing, genetic information processing, human diseases, metabolism, and organismal systems, among which 257 genes were predicted to be associated with human diseases; the *X*- and *Y*-axes indicate gene count and KEGG pathways, respectively. **(C)** CAZyme annotation results of the genome of strain W2469 revealed that it contains seven carbohydrate-active enzyme families. The most abundant families were glycosyl transferases (344 genes) and glycoside hydrolases (317 genes).

COG functional annotation showed that 3,848 protein-coding genes (82.38% of the total protein-coding genes) of strain W2469 were assigned to 24 functional categories, which were mainly enriched in material metabolism (amino acid transport and metabolism, inorganic ion transport and metabolism, coenzyme transport and metabolism, carbohydrate transport and metabolism, lipid transport and metabolism, secondary metabolites biosynthesis, transport and catabolism, energy production and conversion, and nucleotide transport and metabolism), transcription, and signal transduction mechanisms (**Figure 4A**). A small number of genes were annotated to categories such as cell cycle control/cell division/chromosome partitioning, mobilome:prophages/transposons, and defense mechanisms.

KEGG database annotation mapped 2,863 protein-coding genes (61.29% of the total protein-coding genes) to six functional categories, mainly including cellular processes, environmental information processing, genetic information processing, human diseases, metabolism, and organismal systems. Among the annotated genes, 257 were predicted to be associated with human diseases, mainly cancer and neurodegenerative diseases, while genes related to antibiotic resistance and infectious diseases accounted for a high proportion (**Figure 4B**).

#### 4.6.2 CAZyme annotation

CAZy database annotation identified 800 genes encoding carbohydrate-active enzymes in the W2469 genome, covering seven major CAZyme families. The most abundant families were glycosyl transferases (GTs, 344 genes) and glycoside hydrolases (GHs, 317 genes), accounting for more than 80% of total carbohydrate-active enzymes. The remaining genes were annotated to carbohydrate-binding modules (CBMs), carbohydrate esterases (CEs), auxiliary activities (AAs), polysaccharide lyases (PLs), and dockerin domain (Dockerin) (**Figure 4C**). These enzymes participate in the degradation, synthesis, and modification of diverse carbohydrates, which is critical for nutrient utilization and host adaptation.

### 4.7 Niche adaptive and AMR-related genomic features of strain W2469

#### 4.7.1 Virulence factors

**Figure 5:**
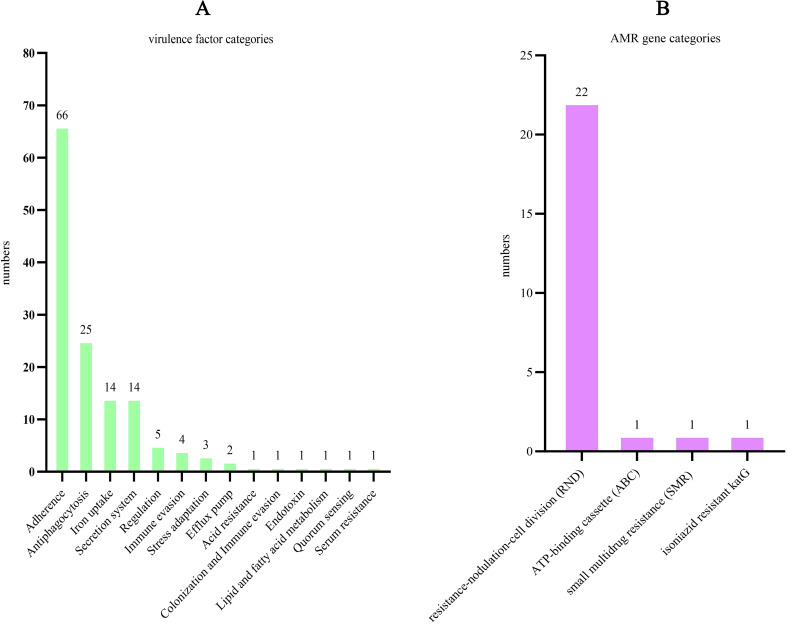
Niche adaptation and AMR-related gene analysis of *Pseudomonas promysalinigenes* W2469. **(A)** Distribution of virulence factor categories; **(B)** distribution of antibiotic resistance gene categories.

VFDB database prediction identified 139 niche adaptation-related homologous factors in the W2469 genome, most of which showed 60%–85% amino acid identity with known reference sequences. These factors were classified into 14 functional categories (**Figure 5A**), with adhesion and colonization-related factors being the most abundant (66 genes), followed by immune evasion-related factors (25 genes) and iron uptake factors (14 genes).

Key virulence factor homologous genes of strain W2469 and their functional annotations are shown in **Table 5**. Adhesion-related virulence factors mainly included flagella (46 genes) and type IV pili (18 genes), which are critical for bacterial motility, chemotaxis, and initial adhesion to host cells. Anti-phagocytosis factors mainly included alginate biosynthesis and regulation genes (22 genes), which contribute to biofilm and capsule formation. Iron uptake factors included pyoverdine and pyochelin siderophore system genes (14 genes), which are essential for iron acquisition in the iron-limited host environment. In addition, the strain contained virulence factor homologous genes related to the type VI secretion system (T6SS), the GacS/GacA two-component quorum sensing system, and stress adaptation (e.g., catalase, superoxide dismutase).

**Table 5:** The key niche adaptation-related homologous factors identified in *Pseudomonas promysalinigenes* W2469.

Virulence factors		Annotation	Numbers
Adherence and colonization module	*flaG*, *fleN*, *fleQ*, *fleR*, *fleS*, *flgA*, *flgB*, *flgC*, *flgD*, *flgE*, *flgF*, *flgG*, *flgH*, *flgI*, *flgJ*, *flgK*, *flgL*, *flgM*, *flgN*, *flhA*, *flhB*, *flhF*, *fliA*, *fliC*, *fliD*, *fliE*, *fliF*, *fliG*, *fliH*, *fliI*, *fliJ*, *fliK*, *fliL*, *fliM*, *fliN*, *fliO*, *fliP*, *fliQ*, *fliR*, *fliS*, *fliT*, *motA*, *motB*, *motC*, *motD*, *motY*	Flagella	46
	*fimV*, *pilA*, *pilB*, *pilC*, *pilD*, *pilE*, *pilF*, *pilM*, *pilN*, *pilP*, *pilQ*, *pilT*	Type IV pili biosynthesis	12
	*chpA*, *chpC*, *pilG*, *pilH*, *pilI*, *pilJ*	Type IV pili twitching motility-related proteins	6
	*cheV*	Polar flagella (*Aeromonas*)	1
Antiphagocytosis and immune evasion module	*alg44*, *alg8*, *algA*, *algC*, *algD*, *algE*, *algF*, *algG*, *algI*, *algJ*, *algK*, *algL*, *algX*	Alginate biosynthesis	13
	*algP/algR3*, *algQ*, *algR*, *algU*, *algW*, *mucA*, *mucB*, *mucD*, *mucP*	Alginate regulation	9
	*gmd-1*	Capsule I (*Burkholderia*)	1
	*gtrB*	LPS glucosylation (*Shigella*)	1
	*manCcore*, *wbpZ*	LPS (*Brucella*)	2
Iron uptake module	*fptA*	Pyochelin receptor	1
	*fpvA*	Pyoverdine receptors	1
	*PvdA*, *pvdE*, *pvdG*, *pvdH*, *pvdL*, *pvdM*, *pvdN*, *pvdO*, *pvdP*, *pvdQ*, *pvdS*, *pvdY*	Pyoverdine	12
Secretion system module	*clpV1*, *hcp1*, *icmF1*, *vgrG1*	Hcp secretion island-1 encoded type VI secretion system (H-T6SS)	4
	*hopAJ2*, *hopJ1*	*P. syringae* TTSS effectors	2
Quorum sensing and regulation module	*hdtS*	Acylhomoserine lactone synthase	1
	*GacA*, *gacS*	GacS/GacA two-component system	2
	*csrA*	Carbon storage regulator A (*Legionella*)	1
	*bfmR*	Two-component system (*Acinetobacter*)	1
	*bvgA*	Two-component system (*Bordetella*)	1
Stress adaptation module	*katG*	Catalase–peroxidase (*Mycobacterium*)	1
	*kata*	Catalase (*Neisseria*)	1
Stress adaptation module	*sodCI*	SodCI (*Salmonella*)	1
	*Icl*	Isocitrate lyase (*Mycobacterium*)	1
Other virulence modules	*acrB*	Efflux pump	1
	*kdsA*	Endotoxin	1
	*rmlC*	Serum resistance	1
	*ureG*	Urease (*Helicobacter*)	1

#### 4.7.2 Antibiotic resistance genes and phenotype correlation

CARD database analysis identified 25 AMR homologous genes in the W2469 genome, 13 of which showed <70% amino acid identity with canonical AMR genes in the CARD database (**Table 6**). These genes belonged to four AMR gene families: resistance–nodulation–cell division (RND), ATP-binding cassette (ABC), small multidrug resistance (SMR), and isoniazid-resistant katG (**Figure 5B**). Among these, 24 genes conferred resistance via antibiotic efflux, and only one gene (katG) acted via antibiotic target alteration. Most efflux-related resistance genes (22 genes) belonged to the RND efflux pump family, the primary intrinsic resistance mechanism in *Pseudomonas* species.

**Table 6:** The antibiotic resistance genes identified in *Pseudomonas promysalinigenes* W2469.

ARO term	AMR gene family	Drug class	Resistance mechanism		% Identity
*YajC*	Resistance–nodulation–cell division (RND) antibiotic efflux pump	Fluoroquinolone antibiotic, cephalosporin, glycylcycline, penicillin β-lactam, tetracycline antibiotic, oxazolidinone antibiotic, glycopeptide antibiotic, rifamycin antibiotic, phenicol antibiotic, disinfecting agents and antiseptics	Antibiotic efflux		90.18
*MexT*	Resistance–nodulation–cell division (RND) antibiotic efflux pump	Fluoroquinolone antibiotic, diaminopyrimidine antibiotic, phenicol antibiotic	Antibiotic efflux		84.01
*MexK*	Resistance–nodulation–cell division (RND) antibiotic efflux pump	Macrolide antibiotic, tetracycline antibiotic	Antibiotic efflux		81.86
*MexW*	Resistance–nodulation–cell division (RND) antibiotic efflux pump	Macrolide antibiotic, fluoroquinolone antibiotic, tetracycline antibiotic, phenicol antibiotic, disinfecting agents and antiseptics	Antibiotic efflux		81.36
*CpxR*	Resistance–nodulation–cell division (RND) antibiotic efflux pump	Macrolide antibiotic, fluoroquinolone antibiotic, monobactam, aminoglycoside antibiotic, carbapenem, cephalosporin, penicillin β-lactam, tetracycline antibiotic, peptide antibiotic, aminocoumarin antibiotic, diaminopyrimidine antibiotic, sulfonamide antibiotic, phenicol antibiotic	Antibiotic efflux		81.25
*rsmA*	Resistance–nodulation–cell division (RND) antibiotic efflux pump	Fluoroquinolone antibiotic, diaminopyrimidine antibiotic, phenicol antibiotic	Antibiotic efflux		78.69
*OprN*	Resistance–nodulation–cell division (RND) antibiotic efflux pump	Fluoroquinolone antibiotic, diaminopyrimidine antibiotic, phenicol antibiotic	Antibiotic efflux		78.1
*TriC*	Resistance–nodulation–cell division (RND) antibiotic efflux pump	Disinfecting agents and antiseptics	Antibiotic efflux		74.43
*MuxB*	Resistance–nodulation–cell division (RND) antibiotic efflux pump	Macrolide antibiotic, monobactam, tetracycline antibiotic, aminocoumarin antibiotic	Antibiotic efflux		72.28
*OpmH*	Resistance–nodulation–cell division (RND) antibiotic efflux pump	Disinfecting agents and antiseptics	Antibiotic efflux		71.97
*MexE*	Resistance–nodulation–cell division (RND) antibiotic efflux pump	Fluoroquinolone antibiotic, diaminopyrimidine antibiotic, phenicol antibiotic	Antibiotic efflux		71.74
*mexN*	Resistance–nodulation–cell division (RND) antibiotic efflux pump	Phenicol antibiotic	Antibiotic efflux		71.43
*OprM*	Resistance–nodulation–cell division (RND) antibiotic efflux pump	Macrolide antibiotic, fluoroquinolone antibiotic, aminoglycoside antibiotic, carbapenem, cephalosporin, penicillin β-lactam, tetracycline antibiotic, phenicol antibiotic, disinfecting agents and antiseptics	Antibiotic efflux		68.6
*MexA*	Resistance–nodulation–cell division (RND) antibiotic efflux pump	Macrolide antibiotic, tetracycline antibiotic	Antibiotic efflux	68.41	
*adeF*	Resistance–nodulation–cell division (RND) antibiotic efflux pump	Fluoroquinolone antibiotic, tetracycline antibiotic	Antibiotic efflux	67.4	
*soxR*	ATP-binding cassette (ABC) antibiotic efflux pump	Fluoroquinolone antibiotic, cephalosporin, glycylcycline, penicillin β-lactam, tetracycline antibiotic, rifamycin antibiotic, phenicol antibiotic, disinfecting agents and antiseptics	Antibiotic target alteration, antibiotic efflux	66.91	
*MexJ*	Resistance–nodulation–cell division (RND) antibiotic efflux pump	Macrolide antibiotic, tetracycline antibiotic	Antibiotic efflux	65.12	
*MexT*	Resistance–nodulation–cell division (RND) antibiotic efflux pump	Fluoroquinolone antibiotic, diaminopyrimidine antibiotic, phenicol antibiotic	Antibiotic efflux	64.12	
*emrE*	Small multidrug resistance (SMR) antibiotic efflux pump	Aminoglycoside antibiotic	Antibiotic efflux	63.64	
*katG*	Isoniazid-resistant katG	Isoniazid-like antibiotic	Antibiotic target alteration	62.95	
*CRP*	Resistance–nodulation–cell division (RND) antibiotic efflux pump	Macrolide antibiotic, fluoroquinolone antibiotic, penicillin β-lactam	Antibiotic efflux	62.91	
*OpmB*	Resistance–nodulation–cell division (RND) antibiotic efflux pump	Macrolide antibiotic, monobactam, tetracycline antibiotic, aminocoumarin antibiotic	Antibiotic efflux	62.58	
*rsmA*	Resistance–nodulation–cell division (RND) antibiotic efflux pump	Fluoroquinolone antibiotic, diaminopyrimidine antibiotic, phenicol antibiotic	Antibiotic efflux	60.66	
*MexV*	Resistance–nodulation–cell division (RND) antibiotic efflux pump	Macrolide antibiotic, fluoroquinolone antibiotic, tetracycline antibiotic, phenicol antibiotic, disinfecting agents and antiseptics	Antibiotic efflux	60.53	
*TriB*	Resistance–nodulation–cell division (RND) antibiotic efflux pump	Disinfecting agents and antiseptics	Antibiotic efflux	59.42	

Thirteen out of 25 AMR homologous genes showed <70% amino acid identity with canonical reference sequences in the CARD database.

The detailed information of the antibiotic resistance genes in the W2469 genome, including the ARO term, AMR gene families, associated antibiotic class, and resistance mechanism, is shown in **Table 6**. These AMR homologous genes are predicted to be associated with potential resistance to multiple classes of antibiotics, including fluoroquinolones, β-lactams (monobactams, carbapenems, penicillins), macrolides, aminoglycosides, phenicols, and tetracyclines. Notably, no acquired antibiotic resistance genes (e.g., β-lactamase genes, aminoglycoside-modifying enzyme genes) were detected in the genome of W2469, and all identified resistance genes were intrinsic genes of the bacterium.

PorinPredict analysis indicated that the *oprD* porin gene in W2469 exhibited low similarity (<40%) to 15 known *oprD* genotypes. Downregulating or losing the expression of the *oprD* gene may be one of the mechanisms contributing to the intermediate susceptibility of W2469 to meropenem.

#### 4.7.3 Association of MGEs with AMR genes and virulence factors

Database analysis predicted 5 prophages, 5 genomic islands, and 18 insertion sequences (mainly *IS*66 and *IS*3 families) in the W2469 genome; no plasmids were identified. Among the 25 antimicrobial resistance genes and 139 virulence factors detected, comparative annotation showed that only one virulence factor (*algU*, related to anti-phagocytosis and immune evasion) was located on an 11-kb GI. No antimicrobial resistance genes or other virulence factors were found in the remaining MGEs.

#### 4.7.4 Type III secretion system analysis

Prediction using EffectiveT3 2.0 initially identified 659 genes with putative secretion signals. However, subsequent genomic annotation and comparative analysis confirmed that these genes were not related to the T3SS. They mainly encoded housekeeping proteins, metabolic enzymes, transporters, flagellar components, and type IV pilin proteins, not T3SS structural subunits or effector proteins. Furthermore, whole-genome comparison against the VFDB and a *Pseudomonas* T3SS reference dataset confirmed that strain W2469 lacks a complete T3SS gene cluster. Notably, strain W2469 lacks a complete T3SS gene cluster and canonical T3SS effector genes (e.g., *exoS*, *exoT*, *exoU*, *exoY*) that are core virulence elements in pathogenic *P. aeruginosa*. This indicates that the adaptive strategy of this rare environmental species in the human host is distinct from the classic cytotoxic paradigm of clinical *P. aeruginosa*.

#### 4.7.5 Pan-genome analysis and clinical-specific genes

Pan-genome analysis of W2469 and three environmental rice rhizosphere strains showed the following: total genes (0% ≤ strains ≤ 100%): 6,193; shell genes (15% ≤ strains ≤ 95%): 2,224; and core genes (99% ≤ strains ≤ 100%): 3,969. A total of 571 clinical-specific genes unique to W2469 were identified. Functional annotation showed that these genes are potentially closely associated with adaptation to the human biliary microenvironment. Key clinical-specific adaptive genes include the following:

1. Hypoxic survival: *cydA*, *cydB*, and *cydX* encoding cytochrome bd oxidase for anaerobic respiration.
2. Biliary adhesion: Type 1 fimbriae genes (*fimA*, *fimC*, *fimD*) and flagellar genes (*fliD*, *flgL*, *flaG*) that mediate colonization of epithelium and gallstones.
3. Stress resistance: DNA repair genes (*radC*, *umuCD*) and heavy metal/toxin resistance genes (*chrA*, *arsB*, *merA*).
4. Nutrient utilization: Polyamine, amino acid, and iron transporters (*potABCD*, *hisJQPH*, *fepBD*) for host nutrient uptake.
5. Mobile genetic elements: Multiple transposases (*IS*5/*IS*3 families), integrases (*fimB*), and phage-related genes.

#### 4.7.6 Promysalin biosynthetic gene cluster

No complete promysalin biosynthetic gene cluster was detected in the clinical isolate W2469. BLASTn alignment against the promysalin BGC of the environmental type strain RW10S1 only identified fragmented homologous segments with <60% sequence identity, lacking core biosynthetic and regulatory genes. This suggests adaptive gene loss during the transition from the plant rhizosphere niche to the human biliary tract: environmental strains retain promysalin for interspecific competition in the rhizosphere, while the clinical isolate has discarded this environment-specific function to prioritize host adaptation.

## 5 Discussion

This study reports the first worldwide clinical isolate of *P. promysalinigenes* (strain W2469) from a human bile specimen of a patient with pathologically confirmed acute suppurative cholecystitis, expanding the known ecological niche of this species from the plant rhizosphere to the human clinical setting. Three core findings from our empirical data form the basis of this work: 1) conventional clinical identification methods (MALDI-TOF MS, VITEK 2 biochemical assay, 16S rRNA sequencing) consistently misidentified this rare species, while WGS-based ANI (98.8%), dDDH (91.1%), and cgSNP phylogeny provided definitive species-level identification; 2) the strain exhibits a clear narrow-spectrum AMR phenotype (resistance to aztreonam and ticarcillin/clavulanic acid, intermediate susceptibility to meropenem) despite harboring 13 AMR homologous genes with <70% amino acid identity to canonical reference sequences in the CARD database; 3) the strain encodes 139 niche adaptation-related homologous factors (60%–85% sequence identity to known references) and 571 clinical-specific unique genes, with complete loss of the environment-specific promysalin biosynthetic gene cluster, indicating niche-specific adaptive evolution from the rhizosphere to the human biliary tract. Notably, the disjunction between low-sequence-identity homologs and clear functional phenotypes observed in this strain directly reveals critical knowledge gaps in the genomic diversity of rare *Pseudomonas* species and uncharacterized adaptive and AMR mechanisms. Collectively, our results, based on empirical phenotypic and genomic data, underscore the inherent limitations of conventional identification methods for rare *Pseudomonas* species and emphasize the indispensable role of WGS-based strategies in the accurate identification and comprehensive genomic characterization of these underrecognized bacteria.

Our empirical data directly demonstrate the failure of conventional clinical identification methods for this rare *P. promysalinigenes* strain: MALDI-TOF MS produced only a 50% low-confidence match to *P. putida* and *P. mosselii*, failing to meet the ≥90% confidence threshold for reliable species identification; the VITEK 2 GN system yielded a high-confidence (99%) false identification as *P. putida*; and 16S rRNA sequencing showed equal 99.79% similarity to *P. urethralis* and *P. juntendi*, unable to resolve species-level identity. These side-by-side comparative data, generated from the same clinical isolate, directly validate the shortcomings of conventional methods for rare *Pseudomonas* species, consistent with previous clinical microbiology reports (Tohya et al., 2022; Arbefeville et al., 2024). This consistent misidentification can be attributed to two data-supported factors: first, *P. promysalinigenes* was validly published in 2022, and its species-specific mass spectrometric and biochemical profiles are not included in the current commercial databases of MALDI-TOF MS and VITEK 2 systems, leading to cross-matching to the closely related *P. putida* group; second, our 16S rRNA sequencing data confirm the high conservation of this gene among closely related *Pseudomonas* species, which limits its discriminatory power for species delineation, as previously described (Klindworth et al., 2013; Church et al., 2020).

In contrast, WGS-derived ANI, dDDH, and cgSNP phylogenetic analyses afforded unambiguous and definitive taxonomic assignment of strain W2469 to *P. promysalinigenes* (Kim et al., 2014; Goris et al., 2007; Liu et al., 2024). The ANI value of 98.8% and dDDH value of 91.1% between strain W2469 and the type strain RW10S1 substantially exceeded the established thresholds for intraspecies affiliation, and cgSNP phylogeny demonstrated robust clustering with RW10S1 at 100% bootstrap support. These findings validate WGS as the gold-standard methodology for the accurate identification of novel and rare *Pseudomonas* species and highlight the urgent need for continuous curation and expansion of commercial diagnostic databases to incorporate phenotypic and genotypic signatures of underrecognized clinical pathogens.

Our antimicrobial susceptibility testing data show that strain W2469 exhibits a narrow-spectrum resistance phenotype: it is susceptible to 10 of 13 tested clinical anti-*Pseudomonas* antibiotics, with confirmed resistance only to aztreonam (MIC ≥64 μg/mL) and ticarcillin/clavulanic acid (MIC ≥128/2 μg/mL) and intermediate susceptibility to meropenem (MIC = 4 μg/mL). *In silico* genomic annotation identified 25 AMR homologous genes associated with this phenotype, 22 of which belong to the RND efflux pump family, the primary intrinsic resistance mechanism in *Pseudomonas* species (Dreier and Ruggerone, 2015). Notably, 13 of these 25 AMR homologs share <70% amino acid identity with canonical resistance genes in the CARD database, yet the strain exhibits a clear AMR phenotype matching the predicted drug classes of these genes. Our MGE analysis found no plasmids and no acquired resistance determinants (e.g., carbapenemases: *blaKPC*, *blaNDM*, *blaVIM*; extended-spectrum β-lactamases: *CTX-M*, *SHV*, *TEM*, *OXA*; aminoglycoside-modifying enzyme genes: *AAC*, *ANT*, *APH*, *AAD*) located on prophages, genomic islands, or insertion sequences; all 25 AMR genes are chromosomally encoded intrinsic factors (Subedi et al., 2018; Sahayarayan et al., 2024). This genetic architecture directly explains the observed narrow resistance phenotype, which is markedly distinct from the MDR/XDR profiles of clinical *P. aeruginosa* strains, which are primarily driven by horizontally acquired resistance loci associated with MGEs (Qin et al., 2022; Zurita et al., 2024).

The observed AMR phenotype of W2469 is directly correlated with its genomic features, supported by our sequencing and susceptibility data: 1) Aztreonam resistance is consistent with the presence of RND efflux pump genes (*cpxR*, *muxB*, *opmB*) whose reference homologs are known to mediate monobactam efflux (Mima et al., 2009; Tian et al., 2016); 2) ticarcillin/clavulanic acid resistance is explained by the combined presence of RND efflux pump genes (*mexA*, *mexB*, *oprM*, *cpxR*, *soxR*) targeting penicillin β-lactams and an intrinsic chromosomally encoded AmpC β-lactamase, which is inherently insensitive to clavulanic acid (Horna et al., 2018; Alimoghadam et al., 2024; Ester et al., 2020; Grosjean et al., 2021); 3) intermediate meropenem susceptibility correlates with the low similarity (<40%) of the strain’s *oprD* porin gene to established functional genotypes, which is known to reduce membrane permeability to carbapenems in *Pseudomonas* species (Sherrard et al., 2022; Ocampo-Sosa et al., 2012). Importantly, all these associated genes are low-identity homologs of canonical resistance genes, and no acquired carbapenemase genes were detected in the genome. This disjunction between low sequence homology and clear phenotypic resistance directly demonstrates that current public databases have significant limitations in annotating functional genes of rare *Pseudomonas* species, and highlights the abundance of uncharacterized AMR mechanisms in the broader bacterial diversity (Dechathai et al., 2025; Subedi et al., 2018; Elbehiry et al., 2022), as we previously emphasized in the Results section.

Our functional genomic annotation data directly reveal multiple adaptive features that support the survival of strain W2469 in the human biliary tract, all of which are anchored to the gene counts and functional categories identified in our study:

Nutrient acquisition and metabolic adaptation: COG and KEGG annotation showed that 82.38% of the strain’s protein-coding genes are assigned to metabolic functions, including amino acid, carbohydrate, and energy metabolism. CAZy annotation identified 800 carbohydrate-active enzyme genes, 82.6% of which are glycosyltransferases (GTs, 344 genes) and glycoside hydrolases (GHs, 317 genes). These loci enable the strain to utilize host-derived polysaccharides and scarce nutrients in the biliary tract, a critical adaptation for persistence in this nutrient-limited host niche (Cantarel et al., 2009; Tatusov et al., 2003; Kanehisa et al., 2017).

Adhesion and colonization determinants: Our VFDB analysis identified 139 niche adaptation-related homologous factors (60%–85% amino acid identity to known references), 66 of which are associated with adhesion and colonization. These include 46 flagellar genes and 18 type IV pilus genes, which are predicted to mediate motility, chemotaxis, and attachment to biliary epithelial cells, consistent with the characteristics of bacteria colonizing the biliary tract (Dasgupta et al., 2003; Craig and Li, 2008).

Stress resistance and host niche adaptation: The strain harbors 25 genes associated with alginate biosynthesis and regulation, which predicted to mediate biofilm formation (Hay et al., 2014; Gao et al., 2024), 14 siderophore system genes for iron acquisition in the iron-depleted biliary environment (Andrews et al., 2003; Bonneau et al., 2020), and a complete T6SS gene cluster (Clemens et al., 2018). These factors are predicted to support survival in the host environment, interbacterial competition, and resistance to host immune stress. Notably, our genomic data confirm that the strain lacks a complete T3SS gene cluster and canonical cytotoxic effector genes (e.g., *exoS*, *exoT*, *exoU*, *exoY*) that are core virulence elements in pathogenic *P. aeruginosa* (Arnold et al., 2009; Galle et al., 2012). This indicates that the adaptive strategy of this rare environmental species in the human host is distinct from the classic cytotoxic paradigm of *P. aeruginosa*, and is instead centered on adhesion, colonization, and stress resistance.

Niche-specific adaptive evolution: Our pan-genome analysis, comparing the clinical isolate with three environmental rhizosphere strains, identified 571 unique genes specific to W2469. These clinical-specific genes are enriched in functions associated with hypoxic survival (*cydA*-*cydB*-*cydX*) (Borisov et al., 2021), oxidative stress resistance (*radC*, *umuC*, *umuD*) (Lima-Noronha et al., 2022), and heavy metal and toxin resistance loci (*chrA*, *ars*, *mer* operons) (Sánchez-Corona et al., 2025), and mobile elements facilitating genomic plasticity (Lang et al., 2025), all of which are relevant to survival in the human biliary tract. In contrast, the strain has completely lost the promysalin biosynthetic gene cluster, which is conserved in environmental strains for rhizosphere-interspecific competition (Das et al., 2023; Li et al., 2011). Based on these genomic data, we propose that this is niche-specific adaptive evolution: the clinical isolate has undergone reductive genome evolution, discarding environment-specific functions while retaining and diversifying genes associated with host niche survival.

Importantly, nearly all of these adaptation-related factors are low-identity homologs of known reference sequences, yet they are enriched in functions directly relevant to biliary tract survival. This directly reflects the limitations of using databases built on known pathogenic bacteria to annotate rare environmental species and highlights the vast diversity of adaptive strategies in non-pathogenic bacteria transitioning to clinical niches.

Several limitations of this study warrant explicit consideration, which also define the boundaries of our conclusions: First, this study describes a single clinical isolate of *P. promysalinigenes*, and all conclusions are limited to the characteristics of this strain; larger-scale epidemiological studies are required to define the prevalence, clinical spectrum, and disease-causing potential of this species in humans. Second, all niche adaptation and AMR-related gene functions are inferred from *in silico* homologous annotation, and the exact roles of these low-identity homologous genes require further *in vitro* and *in vivo* functional validation. Third, the molecular mechanisms of the strain’s adaptation to the human biliary tract and potential host–pathogen interactions remain to be experimentally elucidated. Fourth, antimicrobial susceptibility testing was performed using a commercial clinical panel, and expanded testing is required to fully define the strain’s resistance phenotype.

In summary, our study provides the first worldwide documentation of *P. promysalinigenes* isolated from a human clinical bile specimen, expanding the known ecological niche of this species from the plant rhizosphere to the clinical setting. Our empirical identification data confirm that conventional clinical methods consistently misidentify this rare species, and WGS-based ANI, dDDH, and cgSNP analyses are indispensable for its accurate taxonomic identification. Genomic characterization reveals that strain W2469 harbors low-identity homologs of known AMR and host adaptation factors, yet exhibits clear corresponding phenotypes, and has undergone niche-specific adaptive evolution from the environmental rhizosphere to the human biliary tract. These findings directly reveal critical knowledge gaps in the genomic diversity of rare *Pseudomonas* species and uncharacterized AMR and adaptive mechanisms and provide a foundational genomic resource for understanding the niche transition of environmental bacteria to clinical settings.

## Data Availability

The datasets presented in this study can be found in online repositories. The names of the repository/repositories and accession number(s) can be found below: https://www.ncbi.nlm.nih.gov/genbank/, PX899384, https://www.ncbi.nlm.nih.gov/genbank/, PRJNA1397510, https://www.ncbi.nlm.nih.gov/genbank/, SRR38011177.
